# Bioactive Glass (BG) ICIE16 Shows Promising Osteogenic Properties Compared to Crystallized 45S5-BG

**DOI:** 10.3390/ijms21051639

**Published:** 2020-02-28

**Authors:** Fabian Westhauser, Frederike Hohenbild, Marcela Arango-Ospina, Sarah I. Schmitz, Sebastian Wilkesmann, Leena Hupa, Arash Moghaddam, Aldo R. Boccaccini

**Affiliations:** 1 Center of Orthopedics, Traumatology, and Spinal Cord Injury, Heidelberg University Hospital, Schlierbacher Landstraße 200a, 69118 Heidelberg, Germany; frederike.hohenbild@med.uni-heidelberg.de (F.H.); SarahIsabelle.Schmitz@med.uni-heidelberg.de (S.I.S.); sebastian.wilkesmann@med.uni-heidelberg.de (S.W.); arash.moghaddam@klinikum-ab-alz.de (A.M.); 2 Institute of Biomaterials, University of Erlangen-Nuremberg, Cauerstr. 6, 91058 Erlangen, Germany; marcela.arango@fau.de; 3 Johan Gadolin Process Chemistry Centre, Åbo Akademi University, Biskopsgatan 8, 20500 Turku, Finland; leena.hupa@abo.fi; 4 ATORG - Aschaffenburg Trauma and Orthopedic Research Group, Center for Trauma Surgery, Orthopedics, and Sports Medicine, Klinikum Aschaffenburg-Alzenau, Am Hasenkopf 1, 63739 Aschaffenburg, Germany

**Keywords:** 45S5 bioactive glass, ICIE16 bioactive glass, osteogenic differentiation, proliferation, human mesenchymal stromal cells

## Abstract

The ICIE16-bioactive glass (BG) (48.0 SiO_2_, 6.6 Na_2_O, 32.9 CaO, 2.5 P_2_O_5_, 10.0 K_2_O (wt %)) has been developed as an alternative to 45S5-BG, the original BG composition (45.0 SiO_2_, 24.5 Na_2_O, 24.5 CaO, 6.0 P_2_O_5_ (wt %)), with the intention of broadening the BG sintering window while maintaining bioactivity. Because there is a lack of reports on ICIE16-BG biological properties, the influence of ICIE16-BG on viability, proliferation, and osteogenic differentiation of human mesenchymal stromal cells (MSCs) was evaluated in direct comparison to 45S5-BG in this study. The BGs underwent heat treatment similar to that which is required in order to fabricate scaffolds by sintering, which resulted in crystallization of 45S5-BG (45S5-CBG) while ICIE16 remained amorphous. Granules based on both BGs were biocompatible, but ICIE16-BG was less harmful to cell viability, most likely due to a more pronounced pH alkalization in the 45S5-CBG group. ICIE16-BG outperformed 45S5-CBG in terms of osteogenic differentiation at the cellular level, as determined by the increased activity of alkaline phosphatase. However, granules from both BGs were comparable regarding the stimulation of expression levels of genes encoding for osseous extracellular matrix (ECM) proteins. The addition of therapeutically active ions to ICIE16-BG might further improve its ability to stimulate ECM production and should be investigated in upcoming studies.

## 1. Introduction

During the last decades, significant efforts have been undertaken to optimize the osteogenic properties of synthetic bone substitute materials [1,2]. Bioactive glasses (BGs) have been of specific interest in this field—since the original development of the 45S5-BG composition (in wt %: 45.0 SiO_2_, 24.5 Na_2_O, 24.5 CaO, 6.0 P_2_O_5_) by the group of Hench in the late 1960s, the family of BGs grew rapidly [3,4]. The ions liberated from the 45S5-BG stimulate osteogenic differentiation of bone precursor cells [5,6]. Furthermore, enabled by a cascade of changes on the BG surface resulting in the formation of a carbonate-substituted hydroxyapatite-like (HCA) layer, 45S5-BG bonds to bone and surrounding tissues [7]. Apart from these favorable properties, 45S5-BG also suffers from some limitations, for example, the narrow sintering window leads to crystallization during thermal processing, which is usually required to fabricate scaffolds, thus resulting in a heterogeneous dissolution and reduced bioactivity due to the presence of amorphous and crystalline phases [8,9]. Furthermore, the initial burst release of sodium ions results in a dramatic local pH increase, leading to cell death, at least under in vitro conditions [10,11]. 45S5-BG contains a relatively large amount of sodium ions included originally in the 45S5 composition in order to reduce the melting temperature [11].

Therefore, alternative compositions have been developed such as the ICIE16-BG (in wt %: 48.0 SiO_2_, 6.6 Na_2_O, 32.9 CaO, 2.5 P_2_O_5_, 10.0 K_2_O) that was introduced by Elgayar and coworkers in 2004 [12]. ICIE16-BG shows low tendency towards crystallization when sintered due to a greater sintering window [13,14,15]. Furthermore, the network connectivity (NC) of ICIE16-BG is closer to 45S5-BG compared to other alternatives such as 13-93-BG (in wt %: 53 SiO_2_, 6 Na_2_O, 12 K_2_O, 5 MgO, 20 CaO, 4 P_2_O_5_), which has a higher NC [13]. Higher values of NC result in slower ion exchange and dissolution, which can slow down hydroxyapatite formation [13]. Due to the favorable processing properties, therapeutically active ions can be successfully incorporated in the original ICIE16-BG composition, making ICIE16-BG a candidate for developing non-crystalline (amorphous) scaffolds with controlled degradation behavior to deliver these therapeutically active ions locally [16].

Thus far, the influence of the ICIE16-BG composition on viability, proliferation, and osteogenic differentiation of human bone precursor cells such as mesenchymal stromal cells (MSCs) is unknown. Furthermore, a direct comparison of the biological properties of ICIE16-BG to those of the well-known 45S5-BG has not yet been conducted. Before introducing ICIE16-BG to further fields of applications, its biological properties should be assessed and compared to the established 45S5-BG composition. Therefore, in this study, the impact of ICIE16-BG and 45S5-CBG on viability, proliferation, and osteogenic differentiation of MSCs was evaluated in direct comparison.

The two bioactive glasses were tested in granular form; the granules were fabricated following the same thermal treatment usually required to fabricate scaffolds from BG powders (by sintering), with the result being that ICIE16-BG remained amorphous, whereas 45S5-BG crystallized; hence, a crystallized 45S5-BG (45S5 CBG) was investigated. The intention behind this approach was to generate basic in vitro data of both BGs with the option to transfer both BGs to in vivo settings. When used in vivo, BGs are often applied as scaffolds, for example, 3D engineered constructs that require sintering (a high temperature fabrication process) for their production [8,17,18,19].

## 2. Results

### 2.1. Higher Viability and Proliferation in ICIE16-BG Group

**Figure 1:**
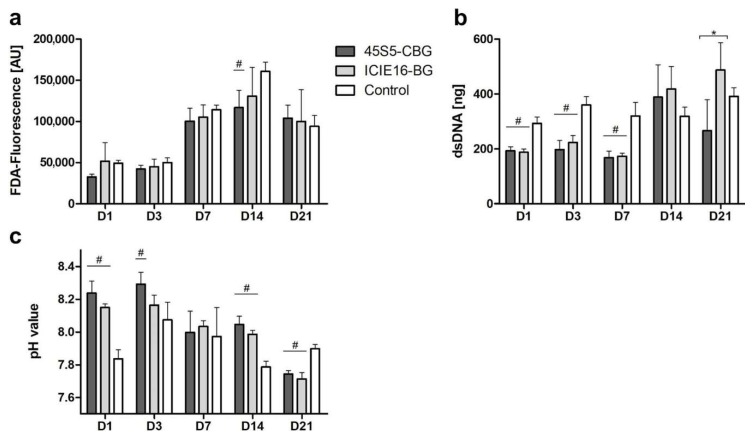
Quantification of cell viability (**a**), proliferation (**b**), and pH (**c**). Values are shown as means with standard deviation. # indicates significant difference to control group and * indicates significance between the bioactive glass (BG) groups being encompassed by brackets.

On day (D)1 to 14, enhanced viability was observed in the ICIE16-BG group compared to 45S5-CBG, with both surpassing the control on D21 (Figure 1a). Proliferation of MSCs exposed to ICIE16-BG increased up to D21 with a significantly higher number of cells compared to the 45S5-CBG group. In the 45S5-CBG group, cell number dropped from D14 to D21 (Figure 1b). On D1 and D14, pH alkalization was significantly higher in the BG groups compared to the control, simultaneously being higher in the 45S5-CBG group in comparison to ICIE16-BG. Only on D21 the pH was significantly lower in the BG groups than in the control group (Figure 1c).

### 2.2. Cell Growth Patterns Were Comparable in the BG Groups

**Figure 2:**
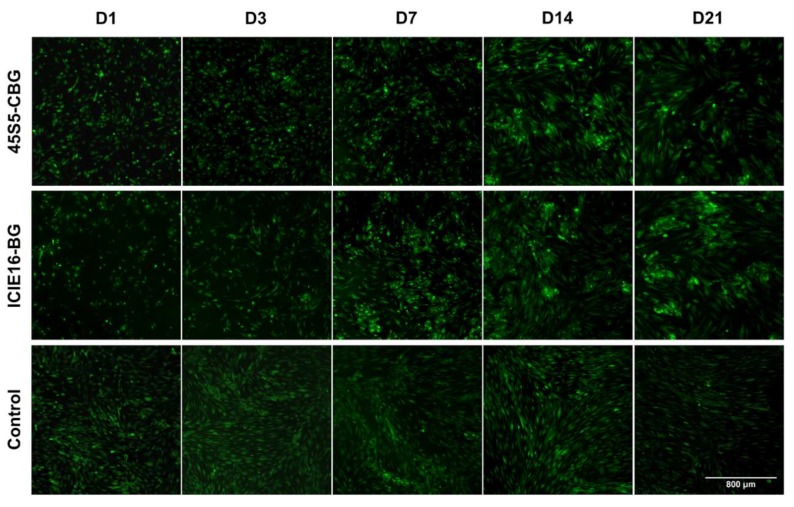
Microscopic visualization of cell viability and growth patterns. Magnification: 40-fold; reference bar refers to 800 µm.

Throughout the entire incubation period, cell density increased in all groups, presenting a consistent cell layer with a higher density in the control group until D14 (Figure 2). From D14 onwards, an altered growth pattern could be observed in the BG groups, showing a high adherence of MSCs to the BG and CBG granules.

### 2.3. BGs Exhibited a Positive Influence on Osteogenic Differentiation

**Figure 3:**
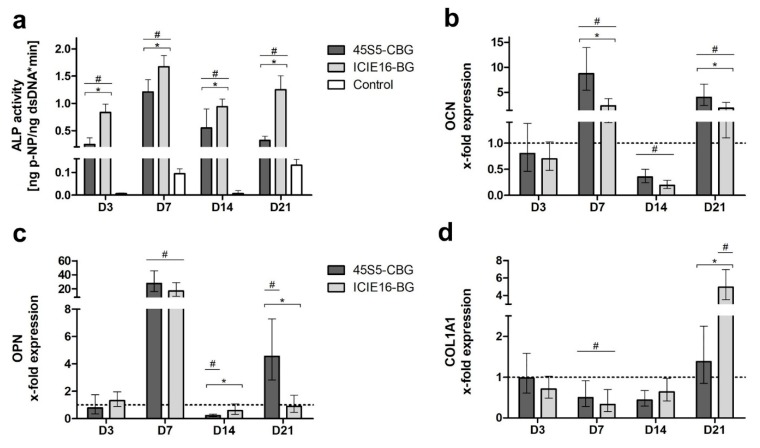
Alkaline phosphatase (ALP) activity (**a**) and expression of osteocalcin (OCN) (**b**), osteopontin (OPN) (**c**), and type I collagen alpha 1 (COL1A1) (**d**). Gene expression was normalized to the control (shown as a dotted line). Values are presented as means with standard deviation. # indicates significant differences to the control group and * indicates significant differences between the BG groups being encompassed by brackets.

Alkaline phosphatase (ALP) activity was significantly higher in the ICIE16-BG group compared to 45S5-CBG on all days. In contrast to the control group, ALP activity was significantly induced in both BG groups from D3 to D14 (Figure 3a). Gene expression levels for osteocalcin (OCN) and osteopontin (OPN) reached a maximum on D7 and a second peak on D21 for both BG and CBG, showing higher values compared to the control, and 45S5-CBG surpassing ICIE16-BG (Figure 3b,c). Type I collagen alpha 1 (COL1A1) expression in the BG and CBG groups remained below the control until D14. On D21, the COL1A1 expression in the ICIE16-BG group significantly exceeded expression levels of both 45S5-CBG and control group (Figure 3d).

## 3. Discussion

Significant effort has been undertaken to improve the biological and mechanical properties of BGs [1]. Amongst several alternatives, the ICIE16-BG exhibits attractive features—compared to 45S5-BG, it shows a lower tendency towards crystallization when sintered due to a greater thermal processing window [13,14,15] while maintaining a NC that is close to 45S5-BG, substantiating favorable bioactivity [13]. Thus far, the impact of ICIE16-BG on viability, proliferation, and osteogenic differentiation of MSCs remains unknown; furthermore, the ICIE16-BG has never been benchmarked to the well-known 45S5-BG composition [10]. In this study, both BGs were compared in the phase assembly achieved after scaffold production for bone tissue engineering by high temperature sintering, with the result being that amorphous ICIE16-BG granules were compared to crystallized 45S5-BG (45S5-CBG).

The release of ions, especially sodium ions, has been made, at least in part, responsible for the in vitro cytotoxicity of BGs [11]—mediated by a burst release of sodium, the local pH rises dramatically followed by cell death. Considering the higher amount of Na^+^ in the composition of 45S5-BG, dissolution of this BG in buffered solutions causes a more pronounced release of Na^+^ ions compared to ICIE16-BG. Even though the Ca^2+^ content in ICIE16-BG is higher than in 45S5 BG, the dissolution profiles of Ca^2+^ ions were similar for both glasses. Regarding the Si^4+^ content, the concentration of released Si^4+^ ions was higher for the ICIE16-BG compared to 45S5 BG; however, the release was faster for 45S5-BG, which is indicative of the superior reactivity of the latter [20,21]. Interestingly, for sintered (crystallized) 45S5-CBG, the release of Si ions showed a similar profile to that of amorphous 45S5-BG [22].

MSCs have been demonstrated as tolerating BG-mediated cytotoxicity better than other cell types, even when being exposed to high concentrations of 45S5-BG [10]. In this study, 45S5-CBG had a higher impact on rising pH values compared to ICIE16-BG. The comparably lower sodium content in ICIE16-BG might explain these findings. Interestingly, the impact on cell viability was not as strong as anticipated because no significant differences could be detected between the granules of BG and CBG groups. However, both groups reduced viability compared to the control group with significant differences between the control group and the 45S5-CBG group on D1 and D14. In this study, MSCs were directly exposed to the BG and CBG granules that were in contact with the cell culture medium. Even though a passivation procedure was conducted to reduce initial bioreactivity of the granules, direct co-culture of cells and BG and CBG had the strongest impact on cell viability compared to other culturing methods such as the indirect culture setting or the trans-well method, with cells only being exposed to ionic dissolution products of the BGs in both cases [18,23,24,25]. It might be concluded that both material types showed biocompatibility in this setting, with ICIE16-BG being less harmful.

During the first week of exposure to BG and CBG, MSCs proliferated less compared to the untreated cells in the control group. From D14 on, cell growth was significantly enhanced by both BG and CBG. When comparing the two granule groups, proliferation was higher in the ICIE16-BG group with significant differences to the 45S5-CBG group on D3 and D21. For the cell culture setting used, it has been described that during the first 5 days of culturing, the MSCs mostly commit to proliferation, whilst osteogenic differentiation starts thereafter [26,27,28]. This is described as the first of three stages of osteogenic differentiation, with early cell differentiation during days 5 to 14 being the second, and formation and mineralization of the ECM during days 14 to 28 being the third and last stage [26]. It must be clarified that these data are based on a setting with cells being cultured in cell culture plastic and not in the presence of biomaterials such as BGs [26]. In the control group, cell number firstly peaked on D3 whereas cell numbers in the BG and CGB groups notably increased from D7 to D14 and finally exceeded the cell number of the control group on D14. It seems, however, that exposure to both BG and CBG granules slowed down initial proliferation, compensated for with increasing incubation time. This behavior might have been a result of the ongoing changes on the BG surface; as described by Greenspan [29], the release of ions from the glass surface starts more or less directly after contact of the BG to the cell culture media (CCM). Within hours, the precipitation of an amorphous calcium phosphate layer takes place, eventually developing into an HCA layer, thus allowing cells to attach after 24-48h [7,29]. According to Greenspan, the attachment of MSCs is then followed by osteogenic differentiation [29]. On the basis of the stages of differentiation, this behavior might explain why the cell proliferation in the BG and CBG groups starts later—cells will proliferate once the HCA layer has formed on the BG surface, a process that takes 2 to 4 days [29]. This means that cell proliferation (encompassing 5 days) will peak between the observation time points D7 and D14 in the used setting. Also, in fluorescence microscopy it was observed that conglomerates of cells grew around the BG and CBG granules from D14 on, indicating that the cells started growing around the granules between D7 and D14 (Figure 3) and not earlier like in BG-free conditions.

The release of therapeutically active ions from BGs exhibit a dose-dependent impact on osteogenic differentiation of bone precursor cells [5,6,18,24,30,31]. Ion release starts immediately after contact of the BGs to the surrounding liquid, in this case, CCM [7,29]. Upon contact to the granules, in this study, the ALP activity, being a differentiation marker during osteoblast development, was significantly increased compared to the control group. When comparing the BGs, ICIE16-BG led to a significantly superior ALP activity over 45S5-CBG. ALP is produced by osteoblasts [32,33,34]; in the used model, MSCs will differentiate into osteoblasts and ALP activity can therefore be used as a direct marker for cellular differentiation [10,33,34]. The physical presence of ICIE16-BG and the ions released from ICIE16-BG seem to be favorable for osteogenic differentiation at a cellular level.

The influence of the BG and CBG on formation of ECM was evaluated by gene expression analysis of OCN, OPN, and COL1A1. OCN and OPN both contribute to ECM mineralization and the mechanical stability of the ECM, for example, by forming tethers between collagen fibrils [35,36]. COL1A1 itself is the most abundant organic compound of the ECM and an important structure protein of the osseous ECM [35]. Although 45S5-CBG significantly stimulates OCN gene expression, no clear superiority of either the granule materials (ICIE16-BG or 45S5-CBG) was found for interaction with OPN and COL1A1 expression levels. It might be concluded that the ICIE16-BG seems to be superior in terms of supporting cellular differentiation (ALP activity), whereas matrix production is stimulated by both granule types without any clear tendency of better behavior. It must be mentioned that the formation of ECM on protein level was not analyzed in this study; therefore, it remains unknown as to how the raised genetic activity levels influenced the actual production of the respective ECM proteins. It has been demonstrated that the biological properties of BGs can be tailored towards specific requirements by incorporation of therapeutically active ions [16,24]. Although ICIE16-BG shows a superior ability to induce osteogenic differentiation on a cellular level in this study, its weaker ability to support the formation of the ECM might be improved by adding appropriate ions to the basic ICIE16-BG composition. Possible candidate ions are, amongst others, zinc, which stimulates protein production in osteoblasts [24,37], or magnesium, which promotes mineralization of the ECM and has been proven to enhance gene expression patterns of OCN and OPN [38,39,40].

## 4. Materials and Methods

### 4.1. BG Production and Characterization

**Figure 4:**
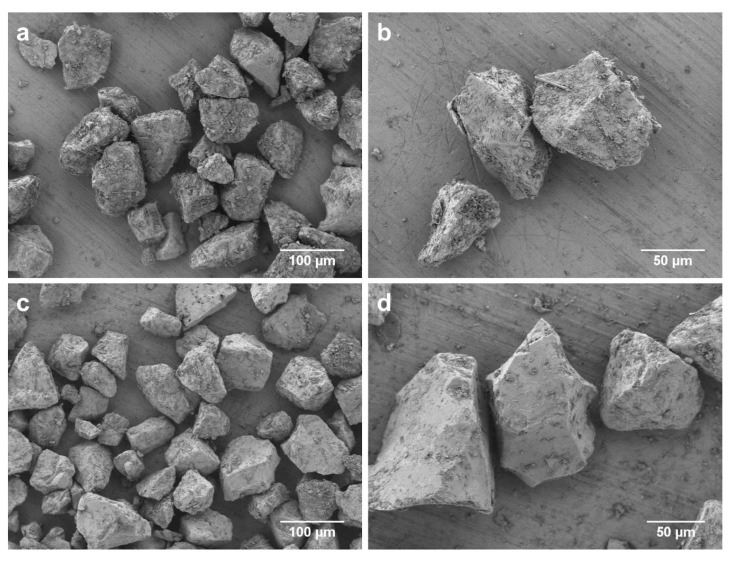
SEM images of the scaffold granules investigated. 45S5-CBG is depicted at a 500-fold magnification in (**a**) and at a 1000-fold magnification in (**b**), whereas ICIE16-BG is shown at a 500-fold magnification in (**c**) and at 1000-fold magnification in (**d**).

Bioactive glasses were prepared according to the melt-quench route using analytical grade reagents, namely, NaCO_3_ (Honeywell Fluka, Steinheim, Germany), K_2_CO_3_ (Alfa Aesar, Erlenbachweg, Germany), CaCO_3_ (Honeywell Fluka, Steinheim, Germany), CaHPO_4_·2H_2_O (Acros Organics, Geel, Belgium), and commercial grade Belgian quartz sand (SiO_2_). ICIE16-BG was melted in Pt crucibles at 1420 °C for 1.5 h, then cast in graphite molds and annealed at 520 °C for 1 h, and a second melting process was performed to ensure homogeneity. 45S5-BG was melted in Pt crucibles at 1400 °C for 1 h and quench rapidly in water. The glasses were crushed using a Jaw Crusher (Retsch, Haan, Germany) and ground into fine powder with a planetary ball mill (Retsch, Haan, Germany). Absolute ethanol (VWR Chemicals, Radnor, PA, USA) was used as a milling medium. For the production of the final granules, suitable sintering profiles to produce porous scaffolds were applied for each glass. ICIE16-BG was sintered at 690 °C for 1.5 h at a heating rate of 2 °C/min, resulting in an amorphous material [14], whereas 45S5-BG was sintered at 1050 °C for 2 h, inducing crystalline phases characteristic of glass-ceramics [8]. Sintered scaffolds were subsequently crushed, ground, and sieved to achieve granules of less than 125 µm, as depicted in Figure 4 (SEM Auriga, Carl Zeiss, Jena, Germany).

In this way, this study was designed to compare the two materials in the phase assembly they are after producing scaffolds for bone tissue engineering (e.g., after undergoing a high temperature sintering process). It is noted that amorphous 45S5-BG was not tested here because it is not possible to fabricate scaffolds by high-temperature sintering that would keep the amorphous character of 45S5-BG (8).

### 4.2. Study Ethics and Cell Origin

MSCs of *n* = 10 patients that underwent surgical treatment at the proximal femur at the Heidelberg Orthopedic University Hospital were harvested and pooled in order to compensate inter-individual differences in cell behavior, as described previously [32]. Prior to collection of the cells, written consent was obtained by all patients. The study was approved (05/10/2015) by the responsible ethic committee of the Medical Faculty of the University of Heidelberg (S-443/2015).

### 4.3. MSC Isolation, Cultivation, and Characterization

MSCs were isolated from freshly obtained bone marrow and prepared as described previously [32,33,34]. In short, cells were washed in phosphate-buffered saline (PBS; Life Technologies, Darmstadt, Germany) and cultivated in expansion medium consisting of 83% Dulbecco’s modified Eagle’s medium (DMEM), 12.5% fetal calf serum (FCS), 2 mM L-glutamine, 1% non-essential amino acids (NEAA), 50 µM β-mercaptoethanol (all Life Technologies), 100 µg/mL penicillin/streptomycin (Biochrom, Berlin, Germany), and 4 ng/mL fibroblast growth factor 2 (Abcam, Cambridge, United Kingdom). Medium was changed after 24 h and subsequently twice per week. Cells were passaged at 80% confluency and used in passage 5 after being pooled in passage 1 according to previously published recommendations [32].

### 4.4. General Experimental Design: Overview

Granules were preconditioned for 24 h in DMEM to reduce their in vitro toxicity. For all groups, each consisting of five biological replicates, MSCs were seeded in 24- and 96-well culture plates (both Nunc, Roskilde, Denmark) and exposed to CCM (89% DMEM, 10% FCS, 1% penicillin/streptomycin) containing BG and CBG granules at a concentration of 2.5 mg/mL. The control consisted of MSCs cultured in BG-free CCM. Media were changed twice weekly.

**Figure 5:**
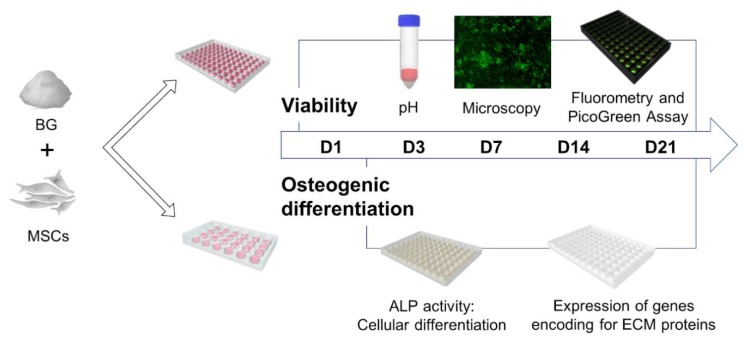
Overview of the experimental design. Mesenchymal stromal cells (MSCs) were co-cultured in direct contact to BG and CBG, respectively. On day (D)1, D3, D7, D14, and D21, viability was evaluated qualitatively and quantitatively via fluorescence microscopy and fluorometry. The content of double-stranded DNA (dsDNA) was assessed by PicoGreen assay and pH values were measured. On D3, D7, D14, and D21, ALP activity and expression of genes encoding for ECM proteins were analysed.

pH alterations and cell viability were measured and visualized on day 1 (D1), 3 (D3), 7 (D7), 14 (D14), and 21 (D21). ALP activity, which is known to increase with the differentiation of MSCs to osteoblasts [33,34], and expression of genes encoding for extracellular matrix (ECM) proteins were evaluated as correlates of osteogenic differentiation on D3, D7, D14, and D21, as schematically depicted in Figure 5.

### 4.5. Evaluation of pH

CCM supernatant was transferred to 15 mL falcon tubes (Merck, Darmstadt, Germany). Opened falcons were incubated at 37 °C and 5% CO_2_ for 5 min to level CO_2_ contents before measuring the pH using a Sartorius PB-11 pH meter (Sartorius, Goettingen, Germany).

### 4.6. Qualitative and Quantitative Assessment of Cell Viability and Proliferation

For visualization of viable cells, staining with fluorescein diacetate (FDA; Sigma-Aldrich, Steinheim, Germany) was performed; FDA freely passes the cell membrane and is intracellularly hydrolyzed to the green-fluorescent fluorescein [32,41]. A total of 100 µL of 10 µg/mL FDA was added to each well and incubated at 37 °C for 5 min. In order to qualitatively evaluate morphology and viability, cells were microscopically analyzed using an Olympus IX-81 inverted fluorescence microscope (Olympus, Hamburg, Germany). For quantification, the staining solution was removed, cells were lysed in 150 µL 0.5% Triton X-100 (Sigma-Aldrich), and fluorescence was measured at 535 nm in a Wallac 1420 Victor microplate reader (Perkin Elmer, Waltham, MA, USA).

In order to quantify MSC proliferation, the dsDNA content, which directly correlates with the number of mononuclear cells, was assessed using the Quant-iT PicoGreen dsDNA Assay Kit (Life Technologies) according to the manufacturer’s instructions.

### 4.7. Evaluation of Alkaline Phosphatase (ALP) Activity as a Marker of Osteoblastic Development

To quantify ALP activity, a para-nitrophenylphosphate (p-NPP)-based colorimetric assay was used. ALP mediates the hydrolysis of p-NPP to para-nitrophenol (p-NP) and phosphate. The amount of accumulating p-NP, a yellow compound whose extinction can be measured at 405 nm, correlates with enzymatic activity of ALP [32,33,34]. In short, cells were lysed with 1% Triton X-100 and stored at −80 °C until further processing. A total of 50 µL of each sample, 50 µL ALP buffer (0.1 M glycine (Carl Roth), 1 mM ZnCl_2_, 1 mM MgCl_2_ (both Merck); pH 10.4), and 100 µL p-NPP (1 mg/mL; Sigma-Aldrich) were incubated for 90 min at 37 °C. The color change was measured in a PHOMO microplate reader (Autobio Diagnostics, Zhengzhou, China) at 405/492 nm. ALP activity of each sample was normalized to the dsDNA content.

### 4.8. qPCR of Gene Activity Encoding for ECM Proteins

Total RNA was isolated via PureLink RNA Mini Kit (Life Technologies) following the manufacturer’s instructions. Complementary DNA (cDNA) synthesis was performed with the High-Capacity RNA-to-cDNA Kit (Life Technologies) according to the manufacturer’s protocol. For real-time quantitative (RT-q) PCR, SYBR Green Master Mix (Life Technologies) was used following the manufacturer’s protocol. Expression of three target genes and one endogenous reference gene was quantified, the primers being listed in Table 1. Relative expression was calculated using the ΔΔCt method, relating each target gene to the reference gene and normalizing the expression to the control group.

### 4.9. Statistics

Statistical analyses were performed using IBM SPSS Statistics (Version 25; IBM, Armonk, NY, USA). Values were tested via one-way ANOVA followed by Bonferroni’s post-hoc test with *p* < 0.05 as the level of significance. Graphs were designed with GraphPad Prism (Version 8.1.0; GraphPad Software, La Jolla, CA, USA). Values are shown as rounded means with standard deviation where applicable.

## 5. Conclusions

In this study, the influence of ICIE16-BG on viability, proliferation, and osteogenic differentiation of MSCs was compared to the well-known 45S5-BG after both BGs underwent thermal treatment similar to the one required to fabricate scaffolds by powder sintering, which led to crystallization of 45S5-BG but not of ICIE16-BG. Granules based on both BGs were biocompatible, however, ICIE16-BG was less harmful to cell viability compared to 45S5-CBG. MSCs grew in direct contact to BG and CBG granules, whereas ICIE16-BG stimulated cell growth in a more pronounced way than 45S5-CBG. In terms of osteogenic properties, ICIE16-BG significantly outperformed 45S5-CBG in terms of ALP activity enhancement as a marker for cellular (osteoblastic) differentiation, whereas the stimulation of gene expression levels encoding for ECM proteins was comparable between the granules based on both BGs. In conclusion, ICIE16-BG is an attractive alternative to 45S5-CBG and should be considered for further applications as scaffold material, especially for in vivo bone tissue engineering. By addition of therapeutically active ions to the ICIE16-BG composition, its ability to stimulate the production of ECM might be improved.

## Figures and Tables

**Table 1:** Primers used for qPCR: glyceraldehyde 3-phosphate dehydrogenase (GAPDH; reference gene), osteocalcin (OCN), osteopontin (OPN), type I collagen alpha 1 (COL1A1).

Gene	Forward (5′→3′)	Reverse (3′→5′)
GAPDH	GCC CAA TAC GAC CAA ATC AGA GA	GAA AGC CTG CCG NGT GAC TAA
OCN	ACC CAG ACA CCA TGA GAC CC	GCT TGG ACA CAA AGG CTG CAC
OPN	GCT AAA CCC TGA CCC ATC TC	ATA ACT GTC CTT CCC ACG GC
COL1A1	GTG GCC TGC CTG GTG AG	GCA CCA TCA TTT CCA CGA GC

## Abbreviations

3D	Three-dimensional
ANOVA	Analysis of variance
BG	Bioactive glass
CBG	Crystallized bioactive glass
CCM	Cell culture media
cDNA	Complementary deoxyribonucleic acid
CO_2_	Carbon dioxide
COL1A1	Type I collagen alpha 1
Ct	Cycle threshold
D	Day
DMEM	Dulbecco’s modified Eagle’s medium
dsDNA	Double stranded deoxyribonucleic acid
ECM	Extracellular matrix
FCS	Fetal calf serum
FDA	Fluorescein diacetate
HCA	Hydroxy carbonate apatite
MSC	Mesenchymal stromal cell
NC	Network connectivity
NEAA	Non-essential amino acids
OCN	Osteocalcin
OPN	Osteopontin
PBS	Phosphate-buffered saline
PCR	Polymerase chain reaction
p-NP	Para-nitrophenol
p-NPP	Para-nitrophenylphosphate
RNA	Ribonucleic acid
SEM	Scanning electron microscopy
